# Association of Shift Work, Sociodemographic Variables and Healthy Habits with Obesity Scales

**DOI:** 10.3390/life14111503

**Published:** 2024-11-18

**Authors:** Javier Tosoratto, Pedro Juan Tárraga López, Ángel Arturo López-González, Daniela Vallejos, Emilio Martínez-Almoyna Rifá, José Ignacio Ramirez-Manent

**Affiliations:** 1 Investigation Group ADEMA SALUD, University Institute for Research in Health Sciences (IUNICS), 07010 Palma, Balearic Islands, Spain; javiertosoratto@gmail.com (J.T.); d.vallejos@eua.edu.es (D.V.); emilio@udemax.com (E.M.-A.R.); joseignacio.ramirez@ibsalut.es (J.I.R.-M.); 2 Faculty of Medicine, UCLM (University of Castilla La Mancha), 02008 Albacete, Castilla-La Mancha, Spain; pjtarraga@sescam.jccm.es; 3 SESCAM (Health Service of Castilla La Mancha), 02008 Albacete, Castilla-La Mancha, Spain; 4 Faculty of Dentistry, University School ADEMA, 07010 Palma, Balearic Islands, Spain; 5 Institut d’Investigació Sanitària de les Illes Balears (IDISBA), Balearic Islands Health Research Institute Foundation, 07010 Palma, Balearic Islands, Spain; 6 Balearic Islands Health Service, 07010 Palma, Balearic Islands, Spain; 7 Faculty of Medicine, University of the Balearic Islands, 07010 Palma, Balearic Islands, Spain

**Keywords:** obesity, shift work, lifestyle habits, Mediterranean diet, exercise, smoking, social class

## Abstract

Background: Shift work has been associated with unhealthy lifestyle habits and a higher prevalence of obesity, which negatively impacts the health of shift workers. The objective of our study was to examine the influence of shift work on obesity, as well as on sociodemographic variables, anthropometric measurements, and lifestyle habits in individuals working this type of schedule. Methods: An observational, cross-sectional, descriptive study involving 53,053 workers from various labour sectors across several Spanish autonomous communities was conducted. It included 31,753 men (17,527 of them working shifts) and 21,300 women (11,281 of them working shifts). The relationship between shift work and obesity was examined, as well as its association with sex, age, social class, education level, smoking, alcohol consumption, sedentary behaviour, and unhealthy diet. Results: Obesity showed higher prevalence and mean values among shift workers across all four formulas used (BMI obesity, WtHR high, CUN BAE obesity, METS-VF high). All variables related to unhealthy lifestyle habits revealed a significantly greater prevalence among shift workers, with high statistical significance (*p* < 0.001). Age, sex, and social class affected the risk of obesity, with a greater prevalence observed in shift workers compared to non-shift workers (*p* < 0.001). Men had a higher risk than women, with an OR ranging from 1.17 (1.12–1.21) for BMI obesity to 7.45 (6.71–8.20) for METS-VF high. Conclusions: Shift workers exhibit a higher prevalence of obesity and unhealthy lifestyle habits, with men at greater risk. The variables that most significantly increase the risk of obesity include age, physical inactivity, low adherence to the Mediterranean diet, and alcohol consumption.

## 1. Introduction

Obesity is one of the most significant and prevalent public health issues worldwide, affecting both developed and developing countries. According to the World Health Organization (WHO), more than 1.9 billion adults were overweight in 2016, with over 650 million classified as obese [1]. This condition is associated with a significant increase in the risk of developing various chronic diseases, including cardiovascular diseases, type 2 diabetes, certain types of cancer, and musculoskeletal disorders [2,3,4]. In addition to the adverse effects on individual health, obesity imposes a considerable economic burden on healthcare systems due to the costs associated with its management and treatment [5,6].

Obesity is a multifactorial condition influenced by a complex interplay of genetic, environmental, and behavioural factors. Identifying and understanding the factors contributing to the development of obesity are essential for formulating effective prevention and treatment strategies [7].

Obesity is more prevalent in middle adulthood, particularly between the ages of 40 and 60, a life stage characterized by changes in body composition and metabolism [8]. A decrease in basal metabolic rate with age, along with a reduction in muscle mass and an increase in adipose tissue, contributes to the development of obesity in older adults. Furthermore, hormonal differences between sexes also play a significant role [9]. Understanding how obesity varies across different life stages is essential for designing age-specific interventions that are more effective.

In terms of sex differences, women tend to have a higher prevalence of obesity than men, especially after menopause. Oestrogens, which have a protective effect against abdominal fat accumulation, significantly decrease after menopause, leading to an increase in central obesity in women [10,11]. Behavioural patterns related to diet and physical activity have also been observed to vary between sexes, which may contribute to the differences noted in obesity prevalence [12]. Recognizing these sex differences is crucial for developing personalized approaches in the prevention and treatment of obesity.

Socioeconomic determinants, such as social class and educational level, are critical factors in the aetiology of obesity. Individuals from lower social classes and with lower educational levels have a higher prevalence of obesity [13]. This is influenced by lower, unhealthy lifestyle practice [14], and limited access to obesity prevention and treatment programs [15,16].

Lifestyle habits also have a significant influence on obesity. Smokers tend to have a lower body weight than non-smokers, which has been attributed to the appetite-suppressing effects of nicotine and the stimulation of thermogenesis [17]. In recent years, as smoking rates have declined in many countries, the prevalence of obesity has been on the rise. This post-cessation weight gain underscores the need to develop post-smoking cessation interventions that also address weight gain prevention in order to maximize health benefits [18]. Alcohol consumption can lead to an increase in food intake, as it may disinhibit eating behaviours, resulting in the consumption of calorie-dense and fatty foods. Several studies have examined the relationship between alcohol consumption and obesity with mixed results. Some studies suggest that moderate alcohol consumption may not be associated with obesity and, in some cases, may even be linked to a lower body mass index (BMI) [19]. However, excessive alcohol consumption and regular intake of high-alcohol-content beverages have consistently been associated with an increased risk of abdominal obesity [20]. Regular physical activity is widely recognized as a key protective factor against obesity. Exercise helps maintain a negative energy balance by increasing energy expenditure and improving body composition by increasing muscle mass and reducing fat mass [21]. Moreover, exercise has beneficial effects on lipid metabolism and insulin sensitivity, contributing to the prevention of obesity and related metabolic diseases [22]. A meta-analysis conducted by Armstrong et al. in 2022 revealed that regular moderate-intensity physical exercise leads to a reduction in visceral adipose tissue, with more pronounced benefits when the exercise performed is of vigorous intensity [23].

Finally, a healthy diet is another crucial lifestyle factor in the prevention of obesity. The Mediterranean diet has been associated with a range of health benefits [24,25], including a protective effect against obesity [26]. Components of the Mediterranean diet, such as its high fibre and antioxidant content along with the quality of fats, contribute to appetite regulation, improved insulin sensitivity, better type 2 diabetes control, and reduced inflammation [27]. Multiple studies have shown that adherence to the Mediterranean diet is inversely related to the risk of obesity and long-term weight gain [28,29,30,31]. The study conducted by Estruch et al. revealed that individuals following a Mediterranean diet supplemented with extra virgin olive oil or nuts had a lower incidence of abdominal obesity compared to those following a low-fat diet [32]. These findings underscore the potential of the Mediterranean diet not only as a tool for obesity prevention but also as a dietary intervention for weight management.

Shift work, particularly night shifts, has been identified as a significant risk factor for obesity. Disruptions to circadian rhythms and sleep patterns can lead to imbalances in metabolism, appetite regulation, and body composition [33]. A study conducted by Qian et al. demonstrated that shift workers have a significantly higher risk of obesity compared to those working conventional hours, which may be explained by the dysregulation of ghrelin, a key hormone in appetite regulation [34]. Moreover, exposure to artificial light during the night can disrupt melatonin levels, impacting metabolic homeostasis [35]. These findings underscore the importance of considering shift work as a relevant factor in the aetiology of obesity and highlight the need to develop interventions that mitigate its adverse effects [36,37,38,39,40]. Despite the abundance of studies examining the relationship between shift work and obesity, little is known about whether this relationship varies according to different anthropometric variables and lifestyle habits [41,42,43].

The objective of our study was to investigate the influence of shift work on obesity, as well as the sociodemographic, anthropometric, and lifestyle variables in individuals who work shifts. This would be useful for establishing public health policies that implement various interventions aimed at improving the health and quality of life of shift workers.

## 2. Materials and Methods

### 2.1. Participants

Our research was based on a cross-sectional, observational, and descriptive study involving 53,053 workers from different autonomous communities in Spain (Balearic Islands, Andalusia, Canary Islands, Valencian Community, Catalonia, Madrid, Castilla-La Mancha, Castilla y León, Basque Country) and various sectors, particularly hospitality, commerce, health, public administration, transportation, industry, and cleaning. The study included 31,753 men (of whom 17,527 worked shifts) and 21,300 women (of whom 11,281 worked shifts). The workers selected for this study were those who participated in the mandatory annual periodic medical examinations conducted by the various participating companies. The selection period spanned from January 2019 to June 2020.

We defined shift work based on the Workers’ Statute, which defines it as ‘any form of organization of teamwork in which workers successively occupy the same jobs according to a certain rhythm, continuous or discontinuous, requiring the worker to provide their services at different hours over a specified period of days or weeks. In our sample, all shift workers rotated between daytime, evening, and night shifts.

Inclusion Criteria:

- Aged between 18 and 69 years.
- Employed under contract with one of the companies participating in this study.
- Consent to participate in this study.
- Permission for the use of their data for epidemiological purposes.

Exclusion Criteria:

- Age below 18 years or above 69 years.
- Non-employment in one of the participating companies.
- Refusal to participate in the research study.
- Refusal to consent to the use of data for epidemiological purposes.
- Lack of a required parameter for scale calculations.
- Presence of any type of cardiometabolic alteration.

**Figure 1:**
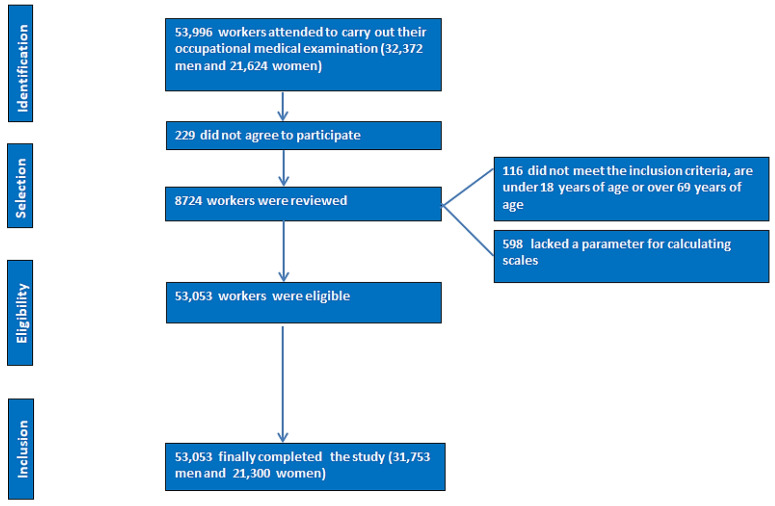
The data from the workers’ flowchart after applying the inclusion criteria.

Figure 1 displays the data from the workers’ flowchart after applying the inclusion criteria.

### 2.2. Determination of Variables

The personnel working in the occupational health departments of the participating companies were responsible for collecting the data needed for this investigation. Anamnesis was one of the methods used for data collection. Information on sociodemographic factors (age, sex, socioeconomic class, and education level) and healthy habits (tobacco use, alcohol consumption, adherence to the Mediterranean diet, and physical activity) was obtained through a comprehensive clinical history.

- Clinical and Anthropometric Measurements: These included height, weight, waist circumference, and systolic and diastolic blood pressure.
- Analytical Measurements: These covered blood glucose levels and lipid profiles.

The independent variables selected were those considered, according to the reviewed literature, to be the most statistically and biologically recommended.

Standardizing the measurement methodologies for these variables was essential to minimize potential biases in the study.

#### 2.2.1. Anthropometric Measurements

Participants’ height and weight were measured while they were standing, wearing only undergarments, with their arms at their sides, and their head and chest aligned. Values were recorded in kilograms and millimetres using a SECA-type scale-measuring apparatus (model: SECA 700 with a capacity of 200 kg and 50 g divisions, to which was added a SECA 220 telescopic height bar with millimetric division and 60–200 cm intervals, SECA, Chino, CA, USA), following international standards for ISAK anthropometric evaluation [44].

Abdominal waist circumference was measured using a SECA model 200 (SECA, Chino, CA, USA) measuring tape placed parallel to the floor, positioned midway between the last rib and the iliac crest while the individual stood relaxed. Hip circumference was also measured in this position by placing the tape measure parallel to the floor at the widest point of the buttocks.

#### 2.2.2. Clinical Measurements

Blood pressure was measured using a calibrated OMRON M3 automatic sphygmomanometer (OMRON, Osaka, Japan). For accurate assessment, the individual sat with his/her back supported by the chair and rested for at least 10 min. Different cuff sizes were available to ensure a proper fit. Three blood pressure measurements were taken consecutively, with one-minute intervals between them. The average of these three readings was used as the final value. Individuals were considered hypertensive if their systolic blood pressure was above 140 mm/Hg, their diastolic blood pressure was above 90 mm/Hg, or if they were undergoing treatment for hypertension.

#### 2.2.3. Analytical Measurements

Blood samples were collected via venipuncture after a 12 h fast. To ensure optimal preservation, samples were processed and stored in a refrigerator for a maximum of 48 to 72 h before being analysed in reference laboratories using standardized techniques. Blood glucose, triglycerides, and total cholesterol levels were measured using enzymatic methods, while HDL cholesterol was measured using precipitation methods. LDL cholesterol was indirectly estimated using the Friedewald formula, provided blood triglyceride levels were below 400 mg/dL; if above this threshold, LDL cholesterol was measured directly [45]. All analytical values were expressed in mg/dL. Patients were classified as dyslipidaemic if their lipid levels exceeded the reference laboratory cutoff points or if they were receiving treatment for dyslipidaemia.

#### 2.2.4. The Overweight and Obesity Scales Determined

- Body Mass Index (BMI) [46]: BMI is calculated by dividing weight in kilograms by height in meters squared. It is categorized as follows: underweight (less than 18.5 kg/m^2^), normal weight (18.5–24.9 kg/m^2^), overweight (25–29.9 kg/m^2^), and obese (30 kg/m^2^ or more).
- Waist-to-Height Ratio (WtHR) [47]: This ratio is determined by dividing the waist circumference by height. Values of 0.50 or higher are considered elevated.
- Clínica Universitaria de Navarra Body Adiposity Estimator (CUN BAE) [48]: The formula for CUN BAE is the following: (CUN BAE)—44.988 + (0.503 × age) + (10.689 × sex) + (3.172 × BMI) − (0.026 × BMI^2^) + (0.181 × BMI × gender) − (0.02 × BMI × age) − (0.005 × BMI^2^ × gender) + (0.00021 × BMI^2^ × age). Male = 0 Female = 1.
- Metabolic score for visceral fat (METS-VF) [49]:
  METS-VF = 4.466 + 0.011 × (Ln(METS-IR))^3^ + 3.239 × (Ln(WHtr))^3^ + 0.319 × (Gender) + 0.594 × (Ln(Age)) where metabolic score for insulin resistance (METS-IR)45 is obtained using the following formula: METS-IR = Ln (2 × glycaemia + triglycerides) × BMI/Ln HDL-c.

Gender was categorized into two dichotomous variables: male and female. Age was calculated by subtracting the date of birth from the date of the medical examination. Of all the validated scales available for assessing obesity, these four were selected because BMI is the most widely used scale in most epidemiological studies; WtHR is a good indicator of abdominal fat; CUN BAE was validated in the Diabetes Care Study as a reliable estimator of body fat; and METS-VF is a good estimator of visceral fat.

Educational level was classified based on the highest level of education completed, with three defined categories: primary studies, secondary studies, and university studies.

Social class was determined according to the guidelines of the Spanish Society of Epidemiology, using the types of occupations listed in the 2011 National Classification of Occupations (CNO-11) [50]. Three social classes were identified:

- Social Class I: Includes university-educated professionals, managers, professional athletes, and artists.
- Social Class II: Consists of intermediate professionals and qualified self-employed individuals.
- Social Class III: Comprises workers with fewer qualifications.

In this study, anyone was considered a smoker if they had not quit smoking for at least one year, or if they had used tobacco products in the previous thirty days.

Adherence to the Mediterranean diet was assessed using a 14-question questionnaire (MEDAS), where each question was scored 0 or 1 point. A total score of nine or more points indicated a high level of adherence [51].

Level of physical activity was measured using the International Physical Activity Questionnaire (IPAQ), a self-administered survey that evaluates physical activity over the previous seven days [52]. The IPAQ classifies physical activity into three categories: low, moderate, and high. The low category does not meet the current physical activity recommendations for health. Therefore, in our study, we considered this as indicating no physical activity.

Alcohol consumption was quantified using standard drinking units, which is the reference method across all levels of care. This method allows for rapid quantification of alcohol intake, easily converted into grams of pure alcohol. In Spain, one standard drinking unit is equivalent to 10 g of alcohol, which corresponds to one serving of wine (100 mL), champagne (100 mL), or beer (200 mL), and half a serving of spirits or mixed drinks (25 mL). Exceeding 35 standard drinking units per week for men and 20 for women poses a significant risk to long-term health [53]. Although we should consider only abstinent individuals as non-drinkers, in our study, we evaluated non-drinkers as those who may consume alcohol occasionally but do not drink daily and do not engage in compulsive drinking—defined as consuming 60 g or more (6 UBEs) for men and 40 g or more (4 UBEs) for women in a single day.

### 2.3. Statistical Analysis

The Kolmogorov–Smirnov test was used to assess the normality of the sample, confirming a normal distribution. To identify possible confounding variables, the corresponding analyses were performed by stratifying the variables included in the model. These analyses showed that none of the variables acted as confounding factors. Quantitative data were analysed using Student’s *t*-test to determine means and standard deviations. The chi-square test (χ^2^) was applied to calculate prevalence when the variables were categorical. A multinomial logistic regression analysis was performed to calculate odds ratios with 95% confidence intervals. The dependent variables included BMI categorized as (underweight, normal weight, overweight, and obesity), waist-to-height ratio categorized as (low, normal, high), CUN BAE categorized as (underweight, normal weight, overweight, and obesity), and METS-VF categorized as (low, normal, high). All statistical analyses were conducted using SPSS software, version 28.0. A *p*-value of less than 0.05 was considered statistically significant.

## 3. Results

Table 1 presents the anthropometric, clinical, analytical, and sociodemographic characteristics of the individuals included in this study. Values are categorized by sex and by the presence or absence of shift work.

All clinical, analytical, and anthropometric parameters showed less healthy values in individuals with shift work, and were consistently worse in men, except for HDL cholesterol. In all cases, the observed differences between workers with and without shifts showed statistical significance, with the exception of height.

The most common age group in both sexes was between 30 and 49 years. The majority of workers, whether in shift work or not, belonged to the most disadvantaged socioeconomic levels (social class III and with elementary education). The percentage of smokers was higher among men with shift work and among women without shifts. Both high adherence to the Mediterranean diet and physical activity were more frequently in both sexes among individuals who did not work shifts. Alcohol consumption was significantly higher among those with shift work.

Table 2 (men) and Table 3 (women) present the prevalence of high values of the four scales assessing excess weight. In all of them, and in both sexes, the values increased with age and decreased in social class. These values were also higher among smokers, sedentary individuals, and those with low adherence to the Mediterranean diet, as well as among those who habitually consumed alcohol. The high percentages values of all scales according to sociodemographic variables and healthy habits were consistently higher in the shift work group, with statistically significant differences in all cases (*p* < 0.001).

Table 4 The results of the multinomial logistic regression analysis are presented, indicating that all variables increase the odds ratio for elevated values across the four scales measuring excess weight. The variables with the greatest association were age, physical inactivity, low adherence to the Mediterranean diet, and alcohol consumption.

## 4. Discussion

Obesity is a major public health issue worldwide, raising significant concern due to its increasing prevalence and association with a wide range of chronic diseases. These include type 2 diabetes (T2DM), cardiovascular diseases (CVD), metabolic syndrome, atherosclerosis, various types of cancer, and a series of diseases secondary to sustained systemic inflammation [54].

The impact of obesity occurs both at the population health level and economically, leading to premature deaths, lost workdays, and costs associated with related diseases. These costs can become unsustainable for healthcare systems, with indirect costs estimated to account for 65% of the total expense, in addition to the direct costs of obesity. This necessitates urgent actions that include both prevention and early diagnosis aimed at curbing obesity. It is essential to address the obesogenic environment in our society by studying all elements that may contribute to its prevalence [55].

Various factors are known to contribute to the development and maintenance of obesity, including both sociodemographic factors and lifestyle choices [56]. This analysis focuses on discussing how shift work, age, sex, social class, educational level, tobacco use, alcohol consumption, physical activity, and adherence to the Mediterranean diet influence the risk of obesity. Additionally, we examine how shift work is associated with unhealthy lifestyle habits that promote the development of obesity. To achieve this, we assessed obesity using four validated scales to determine weight and obesity (BMI, WtHR, CUN BAE, METS-VF), and separated the data by sex, as few studies have investigated the differences in shift work by sex, and the results obtained are contradictory [38,39].

In our study, a total of 53,053 workers from various companies across different autonomous communities in Spain were included. The sample consisted of 31,753 men (of whom 17,527 worked shifts) and 21,300 women (of whom 11,281 worked shifts).

Various studies suggest that shift workers have a higher body mass index (BMI) and prevalence of obesity compared to daytime workers [57]. Among the mechanisms that promote the development of obesity is circadian dysregulation, which affects metabolism and the release of appetite-related hormones, such as leptin and ghrelin [34,58]. This dysregulation occurs when energy intake, wakefulness, and activity take place during the biological night. Circadian disruption also impacts sleep cycles, as daytime sleep alters metabolic reactions and protein dynamics [59]. These irregular sleep patterns common among shift workers, are associated with increased caloric intake and a preference for less healthy foods high in carbohydrates and fats [60].

Age is a key determinant in the development of obesity. The risk of obesity has been observed to increase with age until reaching middle age, after which it may stabilize or even decrease. This can be explained by changes in body composition, decreased muscle mass, reduced basal metabolic rate, and hormonal changes that occur with aging [61]. Additionally, sedentary behaviour tends to increase with age, which also contributes to weight gain [62]. In our study, we observed that the percentage of obese individuals increases with age for both men and women. This progression is so pronounced that, when assessing obesity rates using the CUN BAE formula, the percentage of individuals with obesity exceeds 90% in the age group of 60 to 69 years for both sexes, regardless of shift work. This confirms the association between age and obesity.

When comparing the percentages of obesity between individuals who work shifts and those who do not, the results show a greater number of obese individuals, with statistical significance (*p* < 0.001) across all formulas used in both sexes for shift work. Multinomial logistic regression further supports these findings, showing a higher odds ratio (OR) for shift workers across all formulas used. OR values range from the lowest with BMI, where OR is 1.82 (1.71–1.93) in non-shift workers compared to 2.12 (1.80–2.45) in shift workers, to the highest with the CUN BAE formula for obesity: OR of 9.62 (8.50–10.73) in non-shift workers versus 10.60 (8.91–12.30) in shift workers in the 60–69 age group.

The impact of sex on obesity is complex and multifactorial. Many studies indicate that women exhibit a higher prevalence of obesity compared to men, which may be linked to biological, social, and behavioural factors [63]. There are numerous studies examining the relationship between shift work and obesity; however, it is less well understood whether this relationship varies by sex. Most studies have either included only men [64], only women [65], or have not made comparisons between both sexes [66]. In studies that have compared both sexes, the results have been contradictory [52,67,68,69]. Therefore, given the number of jobs currently involving shift work and its impact on health, it is important to investigate the response of both sexes to shift work.

Our results indicate a higher percentage of obesity among women working shifts compared to their counterparts who do not work shifts, with a high statistical significance (*p* < 0.001) across all variables. When comparing the percentage of elevated obesity values between men and women working shifts, we found that this percentage is higher in men across all variables studied and for all formulas, except in alcohol consumption assessed by the CUN BAE and BMI formulas, where the percentage is higher in women (93.5% in women versus 86.8% in men for CUN BAE and 60.6% in women versus 35.6% in men for BMI). In the case of women, the highest obesity percentages are found using the CUN BAE and WtHR high measurements across all studied variables, with higher percentages among shift workers compared to those who do not work shifts. These differences are significant in all cases (*p* < 0.001). It is noteworthy to highlight the high values of WtHR high, which are indicative of abdominal obesity, representing the phenotype of obesity with the highest risk of morbidity and mortality [70].

In the multinomial logistic regression, when assessing the relationship between sexes, higher odds ratios (OR) are observed for men across all formulas used, with the highest ratio in the METS-VF high (OR5.12 [4.31–5.93]), for shift workers. These findings are consistent with those reported by Son et al. in a Korean population [67].

The relationship between socioeconomic status, educational level, and obesity is well-documented. Generally, a higher prevalence of obesity is observed among individuals of lower socioeconomic classes and those with lower levels of education [71]. This may be attributed to several factors, including limited access to healthy foods, lack of knowledge regarding proper nutrition, and restricted access to physical activity facilities [72]. A lower educational level may also be associated with low-paying jobs that involve irregular hours, thereby increasing the risk of unhealthy behaviours such as poor diet and lack of exercise [41]. Although socioeconomic class and educational level are interrelated, they were studied separately in our analysis. Our results show a higher percentage of obesity as socioeconomic class decreases in both sexes. When comparing these results between shift workers and non-shift workers, we found higher values across all social classes and for all obesity assessment formulas among shift workers. This trend was consistent in both men and women, with a high statistical significance across all social classes (*p* < 0.001). Multinomial logistic regression confirms the association between social class and obesity, both in shift workers and non-shift workers. Shift workers show a higher OR across all formulas used, ranging from 1.59 (1.49–1.69) according to high WtHR to 2.21 (1.81–2.61) according to the CUN BAE formula. A similar trend was observed with educational level. As with social class, multinomial logistic regression indicated that educational level was associated with obesity risk, with higher ORs observed in the shift worker group. In this case, the OR between university education and primary education ranged from 1.58 (1.47–1.59) in the high WtHR formula to an OR of 2.24 (1.70–2.79) for the CUN BAE formula.

When analysing lifestyle habits, we found that obesity shows a higher percentage among smokers in both worker groups and for both sexes, with a statistically significant difference (*p* < 0.001). In the multinomial logistic regression, we observed that smoking increases the risk of obesity across all four formulas evaluated, with a higher odds ratio in shift workers, ranging from 1.23 (1.18–1.29) using BMI to 1.41 (1.30–1.53) with the CUN-BAE formula. The relationship between tobacco consumption and obesity is complex, and the results obtained in our study do not align with those reporting a lower obesity percentage among smokers [73,74]. However, our findings are consistent with other studies indicating that obesity is more prevalent among long-term smokers [75,76].

With regard to alcohol consumption, it has been associated with an increased risk of obesity, although findings are inconsistent. Some studies suggest that moderate alcohol consumption may be linked to a lower body mass index (BMI), while excessive alcohol consumption is associated with an increased risk of obesity and metabolic syndrome [77]. Additionally, alcohol intake may increase appetite and lead to the consumption of unhealthy foods, contributing to excess weight [78]. In our analysis, alcohol consumption was associated with higher percentages of obesity across all formulas evaluated. These differences were statistically significant (*p* < 0.001) across both sexes and for both groups of workers. In the multinomial logistic regression, alcohol consumption yielded one of the highest odds ratios (ORs) across the four formulas used in both worker groups. The OR was higher among shift workers, ranging from 6.12 (5.02–7.23) using high WtHR to 8.40 (7.02–9.79) with high METS-VF, highlighting a strong association with visceral obesity. These results are consistent with other studies identifying a robust relationship between alcohol consumption and obesity [79,80].

Similar to alcohol consumption, physical inactivity shows a strong association with obesity in our findings, with highly significant differences across all formulas (*p* < 0.001), for both sexes and across both worker groups. The highest percentage of physical inactivity was observed in shift workers, with a high level of statistical significance (*p*< 0.001). This aligns with other publications reporting lower levels of physical activity among shift workers [81,82]; however, some authors have noted variations depending on whether shift work was rotational or strictly nocturnal, with night-shift workers exhibiting higher levels of sedentary behaviour and lack of physical activity [83]. Regular physical activity burns calories, improves body composition by increasing muscle mass and reducing body fat [84], improves insulin sensitivity, and regulates appetite hormones [85]. In contrast, a sedentary lifestyle, is associated with weight gain and obesity [86]. The circadian dysregulation experienced by shift workers promotes a decrease in physical activity in both sexes, thus facilitating obesity [87]. In the multinomial logistic regression, not engaging in physical activity is the modifiable variable with the greatest influence on obesity. This increased risk of obesity is observed across all four formulas, raising the risk by over 800% in three of them for shift workers. The influence of sedentarism and physical inactivity on obesity has long been established. A meta-analysis conducted by Silveira et al. in 2022 confirmed this association [88].

The last variable evaluated in our study is adherence to the Mediterranean diet. Similar to the other studied variables, a lack of adherence to the Mediterranean diet is associated with higher obesity, with statistically significant differences across all cases (*p* < 0.001). When analysing the entire sample, we found that the percentage of workers who do not adhere to the Mediterranean diet is significantly higher among shift workers of both sexes. The Mediterranean diet has been widely recognized for its health benefits, including its role in obesity prevention. Adherence to this dietary pattern is associated with reduced long-term weight gain and a lower prevalence of obesity [89]. The components of the Mediterranean diet, such as the monounsaturated fatty acids from olive oil and the antioxidants found in fruits and vegetables, contribute to better regulation of fat metabolism and reduction of inflammation, which may help prevent obesity [90]. Therefore, if the lack of adherence to the Mediterranean diet is a risk factor for obesity, and working shifts significantly increases the lack of adherence to this diet, we can conclude that both are associated and contribute to an increased risk of obesity. The multinomial logistic regression presents it as the third modifiable variable that most increases the risk of obesity.

In various published studies, the relationship between shift work and a healthy diet remains unclear. A study conducted among nursing staff in Spain found no differences in adherence to the Mediterranean diet based on work shifts, with all cases exhibiting either medium or low adherence [91]. On the other hand, a study conducted with healthcare personnel in Poland found that shift-working healthcare professionals had a 50% lower likelihood of adhering to the Mediterranean diet [92]. Our findings align with multiple studies that have found an association between shift work and its negative impact on healthy lifestyle habits [93,94,95]. However, none of these studies include a sample size as large as that of our study.

When comparing the different obesity formulas studied, we observed that the highest percentages of obesity were obtained with the CUN BAE formula, in both sexes and across both worker groups. These percentages were consistently higher in the shift worker group, with high statistical significance (*p* < 0.001). We cannot assert which of these formulas is the most useful for assessing obesity, as all are validated indirect methods that measure different components of body fat. To make such a determination, a DXA (dual-energy X-ray absorptiometry) scan would have been necessary, as it is currently a well-accepted method for evaluating body composition and serves as a standard due to its accuracy and simplicity. In the absence of an objective comparator, such a conclusion cannot be established.

The multinomial logistic regression of the study shows that among the non-modifiable variables, both sex and age are factors that increase the risk of obesity. When comparing the groups of workers, we find that shift workers have a higher odds ratio (OR) for obesity across all the formulas studied, and these differences show high statistical significance (*p* < 0.001). When evaluating the modifiable variables, we find that those that most significantly increase the odds ratio (OR) for obesity are lack of physical activity, alcohol intake, and non-adherence to the Mediterranean diet. As with the other variables, the ORs are higher among shift workers (*p* < 0.001).

Although we cannot modify a person’s sex or age, we can address the modifiable variables, particularly physical activity, alcohol consumption, and diet, which have been shown to significantly increase the risk of obesity. If interventions are implemented at younger ages and tailored motivational actions are taken for each sex, they could reduce the risk of obesity in older age groups, contributing to both social and personal well-being. Further studies are needed to assess whether motivational factors for changing unhealthy lifestyle habits among shift workers vary by sex, in order to identify the most effective interventions for each. It is important to implement interventions in the workplaces of these workers, as the disruption of circadian rhythms may facilitate the increase in obesity. Among these interventions, we suggest the removal of vending machines offering processed pastries and sandwiches, as well as those dispensing sugary and alcoholic beverages. Additionally, implementing health promotion programs and other initiatives to encourage healthier environmental and behavioural changes would be beneficial.

The results of our study demonstrate an undeniable association between shift work and obesity. Shift workers also show a higher percentage of unhealthy lifestyle habits, including smoking, alcohol consumption, sedentary behaviour, and a lack of adherence to the Mediterranean diet (as a healthy dietary pattern). All of these variables have a direct association with obesity. We also found an association between smoking and obesity, which may corroborate the findings of studies with smaller sample sizes.

It is important to recognize that these factors do not operate in isolation but rather interact in various ways to influence the risk of obesity. A person working night shifts may have a higher risk of obesity not only due to the dysregulation of circadian rhythms but also due to a lack of physical activity and the choice of less healthy foods during work shifts. Considering that these variables are modifiable, it is crucial to identify them and take public health and political action to improve the health status of shift workers.

## 5. Strengths and Limitations

Among the strengths of the study is the considerable sample size, which exceeds 53,000 individuals, providing a solid foundation for extrapolating the results to a broader population. This large sample size is essential for enhancing the statistical precision and reliability of the findings.

The origin of the sample from different autonomous communities and various professions may be representative of the Spanish population. In the shift work group, all shift workers rotated between day, evening, and night shifts.

The results obtained, which align with studies published in other countries, allow for the extrapolation of our findings to these populations and validate those obtained from smaller samples.

Another strength of our study is the examination of various variables linking shift work to obesity, differentiated by sex, as there are very few studies in the literature that address this topic, often with contradictory results.

The main limitation of this study is that being a cross-sectional study, it allows for an association between the variables examined and obesity, but causality cannot be established.

The healthy worker effect is a common methodological issue in shift work research, as workers with obesity may be less likely to take up shift work and may be more likely to leave shift work compared to healthy workers. This could potentially underestimate the results.

Since we lack information on the number of years that employees have been working shifts, it is not possible to determine the relationship between the duration of shift work and obesity.

Another limitation of our study is the absence of workplace location as a variable. This omission could potentially lead to differences in results based on specific companies or job positions.

In the assessment of the diet, by only considering adherence to the Mediterranean diet, we lack data on the intake of fast food, sandwiches, pastries, snacks, etc.

## 6. Conclusions

Shift workers face higher obesity rates and unhealthy lifestyle habits, influenced by occupational, demographic, and lifestyle factors. High-risk profiles include older males from lower socioeconomic backgrounds who smoke, consume alcohol regularly, are sedentary, and have low Mediterranean diet adherence.

Key contributors to obesity in shift workers include lack of physical activity (OR 8.83), regular alcohol consumption (OR 8.40), and low Mediterranean diet adherence (OR 4.39). Addressing these factors through public health policies, such as promoting healthy eating, increasing physical activity access, and encouraging Mediterranean diet adoption, is vital for effective obesity prevention and control.

## Figures and Tables

**Table 1:** Characteristics of the workers included in this study.

	Non-Shift Work	Shift Work		Non-Shift Work	Shift Work	
	Men *n* = 14,226	Men *n* = 17,527		Women *n* = 10,019	Women *n* = 11,281	
	Mean (SD)	Mean (SD)	*p*-Value	Mean (SD)	Mean (SD)	*p*-Value
Age (years)	41.2 (10.9)	41.3 (10.5)	0.039	40.0 (10.5)	40.2 (10.3)	0.038
Height (cm)	173.8 (7.1)	173.7 (7.1)	0.219	161.0 (6.6)	161.2 (6.6)	0.075
Weight (kg)	81.5 (14.6)	84.5 (14.4)	<0.001	63.6 (12.8)	68.6 (12.8)	<0.001
Waist (cm)	89.5 (10.5)	90.8 (10.2)	<0.001	74.7 (9.7)	77.6 (10.9)	<0.001
Systolic BP (mmHg)	125.3 (15.7)	126.9 (16.0)	<0.001	114.8 (15.5)	116.1 (15.6)	<0.001
Diastolic BP (mmHg)	75.9 (10.7)	77.2 (11.0)	<0.001	70.3 (10.6)	71.6 (10.8)	<0.001
Total cholesterol (mg/dL)	197.3 (38.4)	201.2 (38.6)	<0.001	192.3 (36.6)	196.9 (37.3)	<0.001
HDL-cholesterol (mg/dL)	50.4 (7.8)	49.7 (7.7)	<0.001	55.0 (9.1)	54.5 (9.2)	<0.001
LDL-cholesterol (mg/dL)	120.9 (37.3)	123.8 (37.6)	<0.001	119.6 (36.9)	123.5 (37.5)	<0.001
Triglycerides (mg/dL)	129.3 (93.7)	136.8 (95.5)	<0.001	87.5 (46.8)	93.6 (51.7)	<0.001
Glucose (mg/dL)	91.9 (26.4)	93.3 (26.4)	<0.001	86.6 (19.0)	87.8 (17.6)	<0.001
	**%**	**%**	***p*-Value**	**%**	**%**	***p*-Value**
18–29 years	16.4	13.8	<0.001	18.6	17.5	0.041
30–39 years	29.3	29.8		31.0	31.3	
40–49 years	29.0	31.3		29.6	30.6	
50–59 years	20.9	20.9		17.9	17.5	
60–69 years	4.4	4.2		2.9	3.1	
Social class I	6.8	8.2	<0.001	11.6	14.6	<0.001
Social class II	20.7	26.6		27.6	37.0	
Social class III	72.5	65.2		60.8	48.4	
Elementary school	69.5	63.8	<0.001	53.7	43.2	<0.001
High school	24.4	28.9		36.2	44.2	
University	6.1	7.3		10.1	12.6	
Non-smokers	67.9	66.0	<0.001	66.3	69.1	<0.001
Smokers	32.1	34.0		33.7	30.9	
Physical inactivity	55.2	67.9	<0.001	40.8	60.7	<0.001
Physical activity	44.8	32.1		59.2	39.3	
NAD Mediterranean diet	58.2	71.5		42.0	63.1	
AD Mediterranean diet	41.8	28.5	<0.001	58.0	36.9	<0.001
Non-alcohol consumption	70.4	63.2	<0.001	85.3	83.5	<0.001
Yes alcohol consumption	29.6	36.8		14.7	16.5	

BP, Blood pressure. HDL, High density lipoprotein. LDL, Low density lipoprotein. AD, Mediterranean diet: Adherence to the Mediterranean diet. NAD, Mediterranean diet: No Adherence to the Mediterranean diet.

**Table 2:** Percentage of high values of different obesity scales according to shift or non-shift work and according to different sociodemographic variables and healthy habits in men.

	Non-Shift Work					Shift Work				
		BMI Obesity	WtHR High	CUN BAE Obesity	METS-VF High		BMI Obesity	WtHR High	CUN BAE Obesity	METS-VF High
Men	*n*	%	%	%	%	*n*	%	%	%	%
18–29 years	2329	7.6	32.1	18.1	0.6	2425	17.3	41.4	32.2	1.5
30–39 years	4174	13.7	47.3	38.0	4.6	5228	25.7	56.8	54.3	6.5
40–49 years	4130	25.3	63.2	64.0	19.3	5477	32.1	68.5	73.5	19.9
50–59 years	2972	35.6	72.8	80.9	34.9	3666	38.7	76.1	86.5	35.9
60–69 years	621	36.1	73.8	90.2	38.6	731	40.8	78.4	93.7	42.8
Social class I	972	20.3	54.9	49.5	13.6	1438	21.0	59.3	58.9	14.5
Social class II	2942	20.5	55.6	53.5	15.9	4669	25.6	63.2	65.7	16.8
Social class III	10,312	25.7	59.6	54.9	18.1	11,420	32.8	64.6	67.1	21.1
Elementary school	9874	26.7	58.8	57.0	18.7	11,169	32.0	63.8	67.6	19.8
High school	3478	21.2	57.8	52.5	16.0	5070	27.2	62.4	65.2	16.3
University	874	19.8	55.0	52.0	13.0	1288	21.9	61.3	61.0	13.8
Non-smokers	9656	19.8	54.7	48.7	12.5	11,567	27.3	59.0	58.7	13.9
Smokers	4570	25.8	61.2	60.6	17.7	5960	31.2	65.4	69.1	18.8
Physical inactivity	7851	39.1	77.9	87.1	28.9	11,899	44.0	78.1	89.2	31.8
Physical activity	6375	4.8	28.9	17.8	2.9	5628	3.2	31.8	22.1	4.6
NAD Mediterranean diet	8275	37.1	75.2	79.2	26.8	12,536	41.7	75.8	83.3	27.5
AD Mediterranean diet	5951	5.9	29.2	17.8	3.9	4991	6.2	31.7	21.2	4.1
Non-alcohol consumption	8996	6.5	35.1	30.3	12.8	12,332	7.0	36.2	54.0	14.6
Yes alcohol consumption	5230	28.5	66.3	79.8	41.2	5195	35.6	76.9	86.8	56.8

BMI, Body mass index. WtHR, Waist to height ratio. CUN BAE, Clínica Universitaria de Navarra Body Adiposity Estimator. AD, Mediterranean diet: Adherence to the Mediterranean diet. NAD, Mediterranean diet: No Adherence to the Mediterranean diet. METS-VF, Metabolic score for visceral fat. *p* < 0.001 in all cases.

**Table 3:** Percentage of high values of different obesity scales according to shift or not shift work and according to different sociodemographic variables and healthy habits in women.

	Non-Shift Work					Shift Work				
		BMI Obesity	WtHR High	CUN BAE Obesity	METS-VF High		BMI Obesity	WtHR High	CUN BAE Obesity	METS-VF High
Women	*n*	%	%	%	%	*n*	%	%	%	%
18–29 years	1869	3.4	9.2	12.5	0.1	1975	6.9	19.7	34.5	0.2
30–39 years	3103	6.8	14.0	21.1	0.4	3530	20.1	26.5	45.8	0.5
40–49 years	2965	14.2	25.2	47.4	2.3	3450	23.2	35.9	65.7	2.9
50–59 years	1791	27.7	37.4	75.1	6.9	1974	30.6	43.9	84.0	7.9
60–69 years	291	31.3	40.2	91.1	10.7	352	37.8	45.5	92.6	11.4
Social class I	1164	3.9	10.7	16.4	0.5	1644	11.8	22.2	39.4	0.9
Social class II	2763	7.8	19.9	25.9	2.9	4175	16.6	32.0	51.5	3.8
Social class III	6092	16.8	24.0	49.2	3.9	5462	30.8	34.6	68.7	5.1
Elementary school	5377	17.0	25.3	49.0	4.0	4871	29.7	38.6	70.0	5.2
High school	3628	9.2	18.2	43.5	2.9	4984	19.5	27.9	52.1	3.8
University	1014	3.5	10.7	30.7	0.5	1426	10.4	22.2	37.9	0.8
Non-smokers	6638	9.6	18.0	32.9	1.5	7794	19.7	28.7	54.2	1.9
Smokers	3381	14.4	23.0	42.1	2.7	3487	24.1	33.2	59.8	3.5
Physical inactivity	4090	31.4	45.2	73.3	5.6	6842	37.6	48.6	84.1	6.8
Physical activity	5929	4.8	4.5	15.3	0.4	4439	6.9	5.9	17.8	0.5
NAD Mediterranean diet	4206	30.5	44.1	67.9	5.4	7115	36.1	47.1	80.1	6.0
AD Mediterranean diet	5813	5.9	4.9	18.0	0.6	4166	7.3	5.8	20.4	0.8
Non-alcohol consumption	8361	3.5	12.0	28.4	0.3	9619	16.2	25.4	51.9	0.6
Yes alcohol consumption	1658	49.9	68.5	92.5	12.6	1662	60.6	69.0	93.5	16.8

BMI, Body mass index. WtHR, Waist to height ratio. CUN BAE, Clínica Universitaria de Navarra Body Adiposity Estimator. METS-VF, Metabolic score for visceral fat. AD, Mediterranean diet: Adherence to the Mediterranean diet. NAD, Mediterranean diet: No Adherence to the Mediterranean diet *p* < 0.001 in all cases.

**Table 4:** Multinomial logistic regression in shift and non-shift workers.

	Non-Shift Work				Yes Shift Work			
	BMI Obesity	WtHR High	CUN BAE Obesity	METS-VF High	BMI Obesity	WtHR High	CUN BAE Obesity	METS-VF High
Non-Shift Work	OR (95% CI)	OR (95% CI)	OR (95% CI)	OR (95% CI)	OR (95% CI)	OR (95% CI)	OR (95% CI)	OR (95% CI)
Women	1	1	1	1	1	1	1	1
Men	1.14 (111–1.17) *	2.85 (2.50–3.21) *	1.84 (1.69–1.99) *	4.19 (3.30–5.09) *	1.33 (1.25–1.41) *	3.19 (2.60–3.79) *	2.13 (1.80–2.46) *	5.12 (4.31–5.93) *
18–29 years	1	1	1	1	1	1	1	1
30–39 yearss	1,12 (1.10–1.15) *	1.16 (1.12–1.20) *	2.11 (1.80–2.42) *	1.23 (1.20–1.27) *	1.19 (1.15–1.23) *	1.32 (1.25–1.40) *	2.16 (2.00–2.33) *	1.30 (1.21–1.40) *
40–49 years	1.23 (1.19–1.27) *	1.29 (1.23–1.35) *	3.69 (3.11–4.27) *	2.03 (1.78–2.28) *	1.33 (1.25–1.41) *	1.49 (1.30–1.68) *	4.02 (3.30–473) *	2.11 (1.81–2.41) *
50–59 years	1.41 (1.35–1.47) *	1.61 (1.50–1.72) *	6.78 (5.80–7.77) *	4.32 (3.60–5.05) *	1.56 (1.41–1.71) *	1.85 (1.60–2.11) *	7.21 (5.98–8.44) *	4.59 (3.90–5.29) *
60–69 years	1.82 (1.71–1.93) *	1.91 (1.78–2.04) *	9.62 (8.50–10.73) *	8.99 (7.50–10.50) *	2.12 (1.80–2.45) *	2.42 (2.01–2.83) *	10.60 (8.91–12.30) *	9.31 (8.01–10.62) *
Social class I	1	1	1	1	1	1	1	1
Social class II	1.27 (1.2–1.33) *	1.26 (1.20–1.32) *	1.48 (1.40–1.57) *	1.30 (1.23–1.37) *	1.39 (1.30–1.49) *	1.38 (1.30–1.47) *	1.52 (1.40–1.64) *	1.39 (1.29–1.50) *
Social class III	1.51 (1.43–1.59) *	1.45 (1.36–1.55) *	1.99 (1.80–2.09) *	1.49 (1.35–1.64) *	1.69 (1.51–1.87) *	1.59 (1.49–1.69) *	2.21 (1.81–2.61) *	1.61 (1.48–1.74) *
University	1	1	1	1	1	1	1	1
High school	1.24 (1.20–1.29) *	1.23 (1.19–1.27) *	1.20 (1.16–1.24) *	1.28 (1.22–1.34) *	1.35 (1.29–1.41) *	1.40 (1.32–1.48) *	1.49 (1.40–1.59) *	1.41 (1.30–1.52) *
Elementary school	1.56 (1.43–1.69) *	1.49 (1.36–1.62) *	1.49 (1.32–1.66) *	1.44 (1.30–11.58) *	1.75 (1.60–1.90) *	1.58 (1.47–1.59) *	2.24 (1.70–2.79) *	1.60 (1.50–1.71) *
Non-smokers	1	1	1	1	1	1	1	1
Smokers	1.12 (1.09–1.15) *	1.14 (1.09–1.20) *	1.40 (1.28–1.52) *	1.32 (1.25–1.39) *	1.23 (1.18–1.29) *	1.26 (1.20–1.32) *	1.41 (1.30–1.53) *	1.38 (1.30–1.47) *
Physical activity	1	1	1	1	1	1	1	1
Physical inactivity	7.65 (6.80–8.51) *	4.85 (4.04–5.65) *	7.45 (6.60–8.31) *	7.21 (6.01–8.41) *	8.83 (7.13–10.54) *	5.69 (4.70–6.69) *	8.23 (7.01–9.45) *	8.39 (7.02–9.77) *
AD Mediterranean diet	1	1	1	1	1	1	1	1
NAD Mediterranean diet	4.11 (3.51–4.72) *	2.23 (1.90–2.56) *	2.29 (2.00–2.59) *	3.12 (2.61–3.63) *	4.39 (3.70–5.09) *	2.89 (2.40–3.39) *	2.50 (2.02–2.98) *	3.60 (2.99–4.21) *
Non-alcohol consumption	1	1	1	1	1	1	1	1
Yes alcohol consumption	6.12 (5.13–7.12) *	5.65 (4.98–6.33) *	6.22 (5.02–7.43) *	7.39 (6.00–8.79) *	8.19 (7.01–9.38) *	6.12 (5.02–7.23) *	7.30 (6.11–8.50) *	8.40 (7.02–9.79) *

BMI, Body mass index. WtHR, Waist to height ratio. CUN BAE, Clínica Universitaria de Navarra Body Adiposity Estimator. METS-VF, Metabolic score for visceral fat. AD, Mediterranean diet: Adherence to the Mediterranean diet. NAD, Mediterranean diet: No Adherence to the Mediterranean diet. (*) *p* < 0.001. *p* < 0.001 in all cases.

## Data Availability

The study data are securely stored in a database that meets all security requirements at the ADEMA-Escuela Universitaria. The Data Protection Officer is Ángel Arturo López González.

## References

[1] World Health Organization (2021). Obesity and Overweight. https://www.who.int/news-room/fact-sheets/detail/obesity-and-overweight.

[2] Sasso E., Baticic L., Sotosek V. (2023). Postprandial Dysmetabolism and Its Medical Implications. Life.

[3] Zhang X., Ha S., Lau H.C., Yu J. (2023). Excess body weight: Novel insights into its roles in obesity comorbidities. Semin. Cancer Biol..

[4] Shumnalieva R., Kotov G., Monov S. (2023). Obesity-Related Knee Osteoarthritis-Current Concepts. Life.

[5] Nagi M.A., Ahmed H., Rezq M.A.A., Sangroongruangsri S., Chaikledkaew U., Almalki Z., Thavorncharoensap M. (2024). Economic costs of obesity: A systematic review. Int. J. Obes..

[6] Okunogbe A., Nugent R., Spencer G., Powis J., Ralston J., Wilding J. (2022). Economic impacts of overweight and obesity: Current and future estimates for 161 countries. BMJ Glob. Health.

[7] Lin X., Li H. (2021). Obesity: Epidemiology, Pathophysiology, and Therapeutics. Front. Endocrinol..

[8] Hales C.M., Fryar C.D., Carroll M.D., Freedman D.S., Ogden C.L. (2018). Trends in Obesity and Severe Obesity Prevalence in US Youth and Adults by Sex and Age, 2007-2008 to 2015-2016. JAMA.

[9] Soysal P., Ates Bulut E., Yavuz I., Isik A.T. (2019). Decreased Basal Metabolic Rate Can Be an Objective Marker for Sarcopenia and Frailty in Older Males. J. Am. Med. Dir. Assoc..

[10] Sheptulina A.F., Antyukh K.Y., Kiselev A.R., Mitkovskaya N.P., Drapkina O.M. (2023). Possible Mechanisms Linking Obesity, Steroidogenesis, and Skeletal Muscle Dysfunction. Life.

[11] Opoku A.A., Abushama M., Konje J.C. (2023). Obesity and menopause. Best. Pract. Res. Clin. Obstet. Gynaecol..

[12] Mattioli A.V., Selleri V., Zanini G., Nasi M., Pinti M., Stefanelli C., Fedele F., Gallina S. (2022). Physical Activity and Diet in Older Women: A Narrative Review. J. Clin. Med..

[13] Aguiló Juanola M.C., López-González A.A., Tomás-Gil P., Paublini H., Tárraga López P.J., Ramírez-Manent J.I. (2023). Influence of tobacco consumption on the values of different overweight and obesity scales in 418,343 Spanish people. Acad. J. Health Sci..

[14] Woodward E.N., Walsh J.L., Senn T.E., Carey M.P. (2018). Positive social interaction offsets impact of low socioeconomic status on stress. J. Natl. Med. Assoc..

[15] Park E., Ko Y. (2021). Socioeconomic Vulnerability Index and Obesity among Korean Adults. Int. J. Environ. Res. Public Health.

[16] Huang Y., Sparks P.J. (2023). Longitudinal exposure to neighborhood poverty and obesity risk in emerging adulthood. Soc. Sci. Res..

[17] Seoane-Collazo P., Diéguez C., Nogueiras R., Rahmouni K., Fernández-Real J.M., López M. (2021). Nicotine’ actions on energy balance: Friend or foe?. Pharmacol. Ther..

[18] Courtemanche C., Tchernis R., Ukert B. (2018). The effect of smoking on obesity: Evidence from a randomized trial. J. Health Econ..

[19] Torres G.G., Siqueira J.H., Martinez O.G.E., Pereira T.S.S., Meléndez J.G.V., Duncan B.B., Goulart A.C., Molina M.D.C.B. (2022). Consumption of alcoholic beverages and abdominal obesity: Cross-sectional analysis of ELSA-Brasil. Cien. Saude Colet..

[20] Wang L.J., Lin C.L., Chen Y.C., Lin C., Shyu Y.C., Chen C.K. (2022). Sex Differences in the Relationship between Excessive Alcohol Consumption and Metabolic Abnormalities: A Community-Based Study in Taiwan. Nutrients.

[21] Pojednic R., D’Arpino E., Halliday I., Bantham A. (2022). The Benefits of Physical Activity for People with Obesity, Independent of Weight Loss: A Systematic Review. Int. J. Environ. Res. Public Health.

[22] Ryan B.J., Schleh M.W., Ahn C., Ludzki A.C., Gillen J.B., Varshney P., Van Pelt D.W., Pitchford L.M., Chenevert T.L., Gioscia-Ryan R.A. (2020). Moderate-Intensity Exercise and High-Intensity Interval Training Affect Insulin Sensitivity Similarly in Obese Adults. J. Clin. Endocrinol. Metab..

[23] Armstrong A., Jungbluth Rodriguez K., Sabag A., Mavros Y., Parker H.M., Keating S.E., Johnson N.A. (2022). Effect of aerobic exercise on waist circumference in adults with overweight or obesity: A systematic review and meta-analysis. Obes. Rev..

[24] Damigou E., Kouvari M., Chrysohoou C., Barkas F., Kravvariti E., Pitsavos C., Skoumas J., Michelis E., Liberopoulos E., Tsioufis C. (2023). Lifestyle Trajectories Are Associated with Incidence of Cardiovascular Disease: Highlights from the ATTICA Epidemiological Cohort Study (2002–2022). Life.

[25] Ramírez-Manent J.I., Belmonte Lomas S., Tárraga Marcos L., López González A.A., Gordito Soler M., Tárraga López P.J. (2023). Analysis of the efficacy of the main dietary patterns in reducing cardiovascular risk. Acad. J. Health Sci..

[26] Bouloukaki I., Daskalaki E., Mavroudi E., Moniaki V., Schiza S.E., Tsiligianni I. (2023). A Dietary and Lifestyle Intervention Improves Treatment Adherence and Clinical Outcomes in Overweight and Obese Patients with Obstructive Sleep Apnea: A Randomized, Controlled Trial. Life.

[27] Celada Roldana C., López Díez J., Cerezuela M.A., Rider F., Tárraga Marcos A., Tárraga López P.J., López González A.A., Ramírez-Manent J.I. (2023). Cardiovascular effects of a nutritional educational intervention in diabetic patients with poor control. Acad. J. Health Sci..

[28] Barber T.M., Kabisch S., Pfeiffer A.F.H., Weickert M.O. (2023). The Effects of the Mediterranean Diet on Health and Gut Microbiota. Nutrients.

[29] Romaguera D., Norat T., Vergnaud A.-C., Mouw T., May A.M., Agudo A., Buckland G., Slimani N., Rinaldi S., Couto E. (2010). Mediterranean dietary patterns and prospective weight change in participants of the EPIC-PANACEA project. Am. J. Clin. Nutr..

[30] Riutord Sbert P., Riutord Fe B., Riutord Fe N., Arroyo Bote S., López González A.A., Ramírez Manent J.I. (2022). Influence of physical activity and mediterranean diet on the values of different scales of overweight and obesity. Acad. J. Health Sci..

[31] Dominguez L.J., Veronese N., Di Bella G., Cusumano C., Parisi A., Tagliaferri F., Ciriminna S., Barbagallo M. (2023). Mediterranean diet in the management and prevention of obesity. Exp. Gerontol..

[32] Estruch R., Ros E., Salas-Salvadó J., Covas M.-I., Corella D., Arós F., Gómez-Gracia E., Ruiz-Gutiérrez V., Fiol M., Lapetra J. (2018). Primary Prevention of Cardiovascular Disease with a Mediterranean Diet Supplemented with Extra-Virgin Olive Oil or Nuts. N. Engl. J. Med..

[33] Hemmer A., Mareschal J., Dibner C., Pralong J.A., Dorribo V., Perrig S., Genton L., Pichard C., Collet T.H. (2021). The Effects of Shift Work on Cardio-Metabolic Diseases and Eating Patterns. Nutrients.

[34] Qian J., Morris C.J., Caputo R., Garaulet M., Scheer F.A.J.L. (2019). Ghrelin is impacted by the endogenous circadian system and by circadian misalignment in humans. Int. J. Obes..

[35] Meléndez-Fernández O.H., Liu J.A., Nelson R.J. (2023). Circadian Rhythms Disrupted by Light at Night and Mistimed Food Intake Alter Hormonal Rhythms and Metabolism. Int. J. Mol. Sci..

[36] Sooriyaarachchi P., Jayawardena R., Pavey T., King N.A. (2022). Shift work and the risk for metabolic syndrome among healthcare workers: A systematic review and meta-analysis. Obes. Rev..

[37] Ahn C.W., Shin S., Lee S., Park H.S., Hong N., Rhee Y. (2022). Association of Shift Work with Normal-Weight Obesity in Community-Dwelling Adults. Endocrinol. Metab..

[38] Brum M.C.B., Dantas Filho F.F., Schnorr C.C., Bertoletti O.A., Bottega G.B., da Costa Rodrigues T. (2020). Night shift work, short sleep and obesity. Diabetol. Metab. Syndr..

[39] Di Tecco C., Fontana L., Adamo G., Petyx M., Iavicoli S. (2020). Gender differences and occupational factors for the risk of obesity in the Italian working population. BMC Public Health.

[40] Sweeney E., Yu Z.M., Dummer T.J.B., Cui Y., DeClercq V., Forbes C., Grandy S.A., Keats M., Parker L., Adisesh A. (2020). The relationship between anthropometric measures and cardiometabolic health in shift work: Findings from the Atlantic PATH Cohort Study. Int. Arch. Occup. Environ. Health.

[41] Shan Z., Li Y., Zong G., Guo Y., Li J., Manson J.E., Hu F.B., Willett W.C., Schernhammer E.S., Bhupathiraju S.N. (2018). Rotating night shift work and adherence to unhealthy lifestyle in predicting risk of type 2 diabetes: Results from two large US cohorts of female nurses. BMJ.

[42] Proper K.I., Jaarsma E., Robroek S.J.W., Schram J.L.D., Boshuizen H., Picavet H.S.J., Verschuren W.M.M., van Oostrom S.H. (2021). The mediating role of unhealthy behavior in the relationship between shift work and perceived health. BMC Public Health.

[43] Hulsegge G., Proper K.I., Loef B., Paagman H., Anema J.R., van Mechelen W. (2021). The mediating role of lifestyle in the relationship between shift work, obesity and diabetes. Int. Arch. Occup. Environ. Health.

[44] Stewart A., Marfell-Jones M., Olds T., Ridder H. (2011). International Standards for Anthropometric Assessment.

[45] Rosales-Rimache J., Apaza-Condori J., Rabanal-Sanchez J., Jari L., Soncco-Llulluy F. (2024). Comparison of the Friedewald and Vujovic methods with the calculated LDL concentration in a biochemical auto-analyzer. Medwave.

[46] Sastre-Alzamora T., Tomás-Gil P., Paublini H., Pallarés L., Ramírez-Manent J.I., López-González A.A. (2023). Relationship between different scales of overweight and obesity and heart age values in 139,634 Spanish workers. Acad. J. Health Sci..

[47] Browning L.M., Hsieh S.D., Ashwell M. (2010). A systematic review of waist-to-height ratio as a screening tool for the prediction of cardiovascular disease and diabetes: 0·5 could be a suitable global boundary value. Nutr. Res. Rev..

[48] Molina-Luque R., Yañez A.M., Bennasar-Veny M., Romero-Saldaña M., Molina-Recio G., López-González Á.A. (2020). A Comparison of Equation Córdoba for Estimation of Body Fat (ECORE-BF) with Other Prediction Equations. Int. J. Environ. Res. Public Health.

[49] Torun C., Ankaralı H., Caştur L., Uzunlulu M., Erbakan A.N., Akbaş M.M., Gündüz N., Doğan M.B., Oğuz A. (2023). Is Metabolic Score for Visceral Fat (METS-VF) a Better Index Than Other Adiposity Indices for the Prediction of Visceral Adiposity. Diabetes Metab. Syndr. Obes..

[50] Domingo-Salvany A., Bacigalupe A., Carrasco J.M., Espelt A., Ferrando J., Borrell C., del Grupo de Determinantes Sociales de la Sociedad Española de Epidemiología (2013). Propuestas de clase social neoweberiana y neomarxista a partir de la Clasificación Nacional de Ocupaciones 2011. Gac. Sanit..

[51] Schröder H., Fitó M., Estruch R., Martínez-González M.A., Corella D., Salas-Salvadó J., Lamuela-Raventós R., Ros E., Salaverría I., Fiol M. (2011). A short screener is valid for assessing Mediterranean diet adherence among older Spanish men and women. J. Nutr..

[52] Craig C.L., Marshall A.L., Sjöström M., Bauman A.E., Booth M.L., Ainsworth B.E., Pratt M., Ekelund U.L., Yngve A., Sallis J.F. (2003). International physical activity questionnaire: 12-country reliability and validity. Med. Sci. Sports Exerc..

[53] Rodríguez-Martos Dauer A., Gual Solé A., Llopis Llácer J.J. (1999). La “unidad de bebida estándar” como registro simplificado del consumo de bebidas alcohólicas y su determinación en España [The “standard drink unit” as a simplified record of alcoholic drink consumption and its measurement in Spain]. Med. Clin..

[54] Savulescu-Fiedler I., Mihalcea R., Dragosloveanu S., Scheau C., Baz R.O., Caruntu A., Scheau A.E., Caruntu C., Benea S.N. (2024). The Interplay between Obesity and Inflammation. Life.

[55] Okunogbe A., Nugent R., Spencer G., Ralston J., Wilding J. (2021). Economic impacts of overweight and obesity: Current and future estimates for eight countries. BMJ Glob. Health.

[56] Jeżewska-Zychowicz M., Gajda R. (2023). Relationship between the Prevalence of Metabolic Disease and Impaired Mobility, Diet, Physical Activity, and Socio-Demographic Characteristics in the Polish Elderly—A Cross-Sectional Study. Life.

[57] McHill A.W., Phillips A.J., Czeisler C.A., Keating L., Yee K., Barger L.K., Garaulet M., Scheer F.A., Klerman E.B. (2017). Later circadian timing of food intake is associated with increased body fat. Am. J. Clin. Nutr..

[58] Molzof H.E., Peterson C.M., Thomas S.J., Gloston G.F., Johnson R.L., Gamble K.L. (2022). Nightshift Work and Nighttime Eating Are Associated With Higher Insulin and Leptin Levels in Hospital Nurses. Front. Endocrinol..

[59] McHill A.W., Melanson E.L., Higgins J., Connick E., Moehlman T.M., Stothard E.R., Wright K.P. (2014). Impact of circadian misalignment on energy metabolism during simulated nightshift work. Proc. Natl. Acad. Sci. USA.

[60] Balieiro L.C., Rossato L.T., Waterhouse J., Paim S.L., Mota M.C., Crispim C.A. (2014). Nutritional status and eating habits of bus drivers during the day and night. Chronobiol. Int..

[61] Malandrino N., Bhat S.Z., Alfaraidhy M., Grewal R.S., Kalyani R.R. (2023). Obesity and Aging. Endocrinol. Metab. Clin. N. Am..

[62] Dogra S., Dunstan D.W., Sugiyama T., Stathi A., Gardiner P.A., Owen N. (2022). Active Aging and Public Health: Evidence, Implications, and Opportunities. Annu. Rev. Public Health.

[63] Dade E., Metz M., Pierre J.L., Rouzier V., Sufra R., Fox E., Preval F., St-Preux S., Zephir J.R., Ariste W. (2022). High prevalence of obesity among women in urban Haiti: Findings from a population-based cohort. Front. Public Health.

[64] Grundy A., Cotterchio M., Kirsh V.A., Nadalin V., Lightfoot N., Kreiger N. (2017). Rotating shift work associated with obesity in men from northeastern Ontario. Health Promot. Chronic Dis. Prev. Can..

[65] Ramin C., Devore E.E., Wang W., Pierre-Paul J., Wegrzyn L.R., Schernhammer E.S. (2015). Night shift work at specific age ranges and chronic disease risk factors. Occup. Environ. Med..

[66] Givens M.L., Malecki K.C., Peppard P.E., Palta M., Said A., Engelman C.D., Walsh M.C., Nieto F.J. (2015). Shiftwork, Sleep Habits, and Metabolic Disparities: Results from the Survey of the Health of Wisconsin. Sleep. Health.

[67] Son M., Ye B.J., Kim J.I., Kang S., Jung K.Y. (2015). Association between shift work and obesity according to body fat percentage in Korean wage workers: Data from the fourth and the fifth Korea National Health and Nutrition Examination Survey (KNHANES 2008–2011). Ann. Occup. Environ. Med..

[68] Wyse C.A., Celis Morales C.A., Graham N., Fan Y., Ward J., Curtis A.M., Mackay D., Smith D.J., Bailey M.E.S., Biello S. (2017). Adverse metabolic and mental health outcomes associated with shiftwork in a population-based study of 277,168 workers in UK biobank. Ann. Med..

[69] Czyż-Szypenbejl K., Mędrzycka-Dąbrowska W. (2024). The Impact of Night Work on the Sleep and Health of Medical Staff-A Review of the Latest Scientific Reports. J. Clin. Med..

[70] Jayedi A., Soltani S., Zargar M.S., Khan T.A., Shab-Bidar S. (2020). Central fatness and risk of all cause mortality: Systematic review and dose-response meta-analysis of 72 prospective cohort studies. BMJ.

[71] Anekwe C.V., Jarrell A.R., Townsend M.J., Gaudier G.I., Hiserodt J.M., Stanford F.C. (2020). Socioeconomics of Obesity. Curr. Obes. Rep..

[72] Gallego A., Olivares-Arancibia J., Yáñez-Sepúlveda R., Gutiérrez-Espinoza H., López-Gil J.F. (2024). Socioeconomic Status and Rate of Poverty in Overweight and Obesity among Spanish Children and Adolescents. Children.

[73] López-González A.A., Ramírez Manent J.I., Vicente-Herrero M.T., García Ruiz E., Albaladejo Blanco M., López Safont N. (2022). [Prevalence of diabesity in the Spanish working population: Influence of sociodemographic variables and tobacco consumption]. An. Sist. Sanit. Navar..

[74] Trovato A., Tsang T., Manem N., Donovan K., Gemoets D.E., Ashley C., Dellon E.S., Tadros M. (2023). The Impact of Obesity on the Fibrostenosis Progression of Eosinophilic Esophagitis in a U.S. Veterans Cohort. Dysphagia.

[75] Choi S.H., Stommel M., Broman C., Raheb-Rauckis C. (2023). Age of Smoking Initiation in Relation to Multiple Health Risk Factors among US Adult Smokers: National Health Interview Survey (NHIS) Data (2006–2018). Behav. Med..

[76] Lenártová P., Gažarová M., Mrázová J., Kopčeková J., Habánová M., Chlebo P., Jančichová K. (2021). Obesity, smoking status and their relationships in selected population groups. Rocz. Panstw. Zakl. Hig..

[77] Åberg F., Färkkilä M. (2020). Drinking and Obesity: Alcoholic Liver Disease/Nonalcoholic Fatty Liver Disease Interactions. Semin. Liver Dis..

[78] Aitken C.M., Jaramillo J.C.M., Davis W., Brennan-Xie L., McDougall S.J., Lawrence A.J., Ryan P.J. (2023). Feeding signals inhibit fluid-satiation signals in the mouse lateral parabrachial nucleus to increase intake of highly palatable, caloric solutions. J. Neurochem..

[79] Åberg F., Byrne C.D., Pirola C.J., Männistö V., Sookoian S. (2023). Alcohol consumption and metabolic syndrome: Clinical and epidemiological impact on liver disease. J. Hepatol..

[80] Park E.J., Shin H.J., Kim S.S., Kim K.E., Kim S.H., Kim Y.R., Chung K.M., Han K.D. (2022). The Effect of Alcohol Drinking on Metabolic Syndrome and Obesity in Koreans: Big Data Analysis. Int. J. Environ. Res. Public Health.

[81] Pepłońska B., Burdelak W., Krysicka J., Bukowska A., Marcinkiewicz A., Sobala W., Klimecka-Muszyńska D., Rybacki M. (2014). Night shift work and modifiable lifestyle factors. Int. J. Occup. Med. Environ. Health.

[82] Bushnell P.T., Colombi A., Caruso C.C., Tak S. (2010). Work schedules and health behavior outcomes at a large manufacturer. Ind. Health.

[83] Loprinzi P.D. (2015). The effects of shift work on free-living physical activity and sedentary behavior. Prev. Med..

[84] Pazzianotto-Forti E.M., Moreno M.A., Plater E., Baruki S.B.S., Rasera-Junior I., Reid W.D. (2020). Impact of Physical Training Programs on Physical Fitness in People With Class II and III Obesity: A Systematic Review and Meta-Analysis. Phys. Ther..

[85] Gonzalez-Gil A.M., Elizondo-Montemayor L. (2020). The Role of Exercise in the Interplay between Myokines, Hepatokines, Osteokines, Adipokines, and Modulation of Inflammation for Energy Substrate Redistribution and Fat Mass Loss: A Review. Nutrients.

[86] Al-Hazzaa H.M., Albawardi N.M. (2021). Obesity, Lifestyle Behaviors, and Dietary Habits of Saudi Adolescents Living in Riyadh (ATLS-2 Project): Revisited after a Ten-Year Period. Life.

[87] Qian J., Morris C.J., Caputo R., Wang W., Garaulet M., Scheer F.A.J.L. (2019). Sex differences in the circadian misalignment effects on energy regulation. Proc. Natl. Acad. Sci. USA.

[88] Silveira E.A., Mendonça C.R., Delpino F.M., Elias Souza G.V., Pereira de Souza Rosa L., de Oliveira C., Noll M. (2022). Sedentary behavior, physical inactivity, abdominal obesity and obesity in adults and older adults: A systematic review and meta-analysis. Clin. Nutr. ESPEN.

[89] Ramírez Gallegos I., Marina Arroyo M., López-González Á.A., Vicente-Herrero M.T., Vallejos D., Sastre-Alzamora T., Ramírez-Manent J.I. (2024). The Effect of a Program to Improve Adherence to the Mediterranean Diet on Cardiometabolic Parameters in 7034 Spanish Workers. Nutrients.

[90] Itsiopoulos C., Mayr H.L., Thomas C.J. (2022). The anti-inflammatory effects of a Mediterranean diet: A review. Curr. Opin. Clin. Nutr. Metab. Care.

[91] Leyva-Vela B., Reche-García C., Hernández-Morante J.J., Martínez-Olcina M., Miralles-Amorós L., Martínez-Rodríguez A. (2021). Mediterranean Diet Adherence and Eating Disorders in Spanish Nurses with Shift Patterns: A Cross-Sectional Study. Medicina.

[92] Wolska A., Stasiewicz B., Kaźmierczak-Siedlecka K., Ziętek M., Solek-Pastuszka J., Drozd A., Palma J., Stachowska E. (2022). Unhealthy Food Choices among Healthcare Shift Workers: A Cross-Sectional Study. Nutrients.

[93] Peñalvo J.L., Mertens E., Muñoz-Cabrejas A., León-Latre M., Jarauta E., Laclaustra M., Ordovás J.M., Casasnovas J.A., Uzhova I., Moreno-Franco B. (2021). Work Shift, Lifestyle Factors, and Subclinical Atherosclerosis in Spanish Male Workers: A Mediation Analysis. Nutrients.

[94] Nea F.M., Pourshahidi L.K., Kearney J.M., Livingstone M.B.E., Bassul C., Corish C.A. (2018). A qualitative exploration of the shift work experience: The perceived effect on eating habits, lifestyle behaviours and psychosocial wellbeing. J. Public Health.

[95] Sugiura T., Dohi Y., Takagi Y., Yoshikane N., Ito M., Suzuki K., Nagami T., Iwase M., Seo Y., Ohte N. (2020). Impacts of lifestyle behavior and shift work on visceral fat accumulation and the presence of atherosclerosis in middle-aged male workers. Hypertens. Res..

